# Palliative radiotherapy to dominant symptomatic lesion in patients with hormone refractory prostate cancer (PRADO)

**DOI:** 10.1186/s13014-019-1209-0

**Published:** 2019-01-10

**Authors:** Jesper Carl, Dirk Rades, Claudia Doemer, Cornelia Setter, Jürgen Dunst, Niels Henrik Holländer

**Affiliations:** 1 Department of Oncology and Palliative Units, Zealand University Hospital, Naestved, Denmark; 2 Department of Radiation Oncology, University of Lübeck, Lübeck, Germany; 3 Department of Radiation Oncology, Christian-Albrechts University Kiel, Kiel, Germany

**Keywords:** Prostate cancer, Metastatic disease, Dominant symptomatic lesion, High-precision radiotherapy, Hypo-fractionation, Feasibility

## Abstract

**Background:**

This study was conducted to investigate a new short-course radiotherapy regimen for patients with metastatic hormone refractory prostate cancer (HRPC) presenting with a dominant debilitating symptom.

**Methods / design:**

This is an international, multi-center single arm prospective feasibility study that aims to include 34 patients with HRPC and a dominant debilitating symptom. The dominant symptomatic lesion will receive 4 × 5 Gy of high-precision radiotherapy, and the most aggressive part of the lesion 4 × 7 Gy using a simultaneous integrated boost technique. Based on advanced magnetic resonance imaging (MRI), an apparent diffusion coefficient (ADC) map will be calculated for the lesion using diffusion weighted imaging sequences. The dominant symptomatic lesion (GTV1) is drawn manually using the information from T2w-MRI and computed tomography scans. The most aggressive part of the dominant lesion (GTV2) is defined by using the ADC map. An auxiliary volume is created including only voxels in the GTV1 that presents with ADC values below 1200 × 10^− 6^ mm^2^/s. The most aggressive part is defined as voxels with an ADC value below the median ADC value. Primary endpoint is feasibility, i.e. proportion of patients who complete radiotherapy with ≥90% of the prescribed dose. Secondary endpoints include dominant symptom score, progression-free survival (freedom from symptoms), overall survival, acute toxicity, quality of life, change in ADC from baseline to end of treatment and 6 months following treatment.

**Discussion:**

If this new radiotherapy regimen proves to be feasible, a prospective randomized phase II/III dose escalation study will be designed in order to improve the outcomes of palliative radiotherapy of symptomatic metastatic HRPC.

**Study status:**

The study is ongoing and will be recruiting patients soon.

**Trial registration:**

clinicaltrials.gov NCT03658434. Initially registered on 30th of July, 2018

## Background and rationale

Prostate cancer is the major cause of cancer death in men living in developed countries. Large autopsy studies found that 35% of the patients with prostate cancer had metastatic disease. The most common metastatic sites include bone (90%), lung (46%), liver (25%) and pleura (21%) [1]. Patients may also present with disabling symptoms from local pelvic disease progression with an incidence of 10–18% or higher in node-positive patients treated with anti-androgen therapy alone [2–4]. Pelvic radiotherapy (RT) can provide effective palliation by contributing to relief of hematuria, pain and other symptoms [5–9].

Metastatic or locally advanced prostate cancer (PC) initially responds well to hormonal manipulation by androgen withdrawal and peripheral androgen blockade. However, such patients have high risk of developing a hormone-refractory prostate cancer (HRPC). Once the tumor has achieved a castration-refractory metastatic stage, treatment options are limited, and the average survival time of these patients is relatively short [10]. Furthermore, HRPC patients are often elderly or very elderly men with significant co-morbidities. Therefore, a short course of radiotherapy would be a desirable option for these patients. However, prostate cancer usually requires comparably high radiation doses, which are generally difficult to administer within very few days when using conventional radiotherapy. Moreover, in case of large treatment volumes, radiotherapy can be associated with significant toxicities, particularly in the rectum and the urinary bladder [11–14].

With modern high-precision radiotherapy techniques such as volumetric modulated arc therapy (VMAT), stereotactic body radiation therapy (SBRT) and stereotactic radiosurgery (SRS), better sparing of the organs at risk and administration of higher radiation doses in a short time can be achieved. A recent review suggested that SBRT and SRS are effective for metastatic prostate cancer [15]. Further improvement of the treatment results may be achieved by using specific diagnostic imaging procedures for treatment planning such as advanced magnetic resonance imaging (MRI) with diffusion weighted imaging (DWI) [16–20]. When using DWI, quantitative measures such as the apparent diffusion coefficient (ADC) were shown to be the most important modality for predicting tumor location and local tumor recurrences larger than 0.4 ml [18–20]. Thus, change of the ADC can be used as a surrogate marker of response [21–25].

The present feasibility study was conducted to investigate a new short-course radiotherapy regimen for patients with metastatic HRPC presenting with a dominant debilitating symptom, for example pain, urinary retention, chronic rectal obstruction or bleeding. The number of metastases is not limited and could be one lesion (if symptomatic), oligo-metastatic disease (up to 5 lesions) or multiple metastases (more than 5 lesions). This new approach will use MRI to verify correspondence between the progressive HRPC lesion (DSL = the dominant symptomatic lesion) and the patient’s dominant symptom. DWI will be used to identify both the lesion and the most aggressive part of the lesion. The DSL can be any symptomatic metastatic lesion. It will receive 4 × 5 Gy of high-precision radiotherapy, and the most aggressive part of the lesion 4 × 7 Gy using a simultaneous integrated boost (SIB) technique. Based on the results of this study, a prospective randomized phase II/III dose escalation study will be designed. Fractions of 7 Gy were recently used for hypofractionated image-guied radiotherapy for oligo-metastatic prostate cancer [26]. Since the Danish standard regimen for palliative treatment is short-course radiotherapy woth four fractions (4 × 5 Gy), four times 7 Gy was selected for the most aggressive part of the DSL in the present study.

### Endpoints of the study

#### Primary endpoint

- Feasibility, i.e. proportion of study participants who complete radiotherapy with ≥90% of the prescribed dose

#### Secondary endpoints

- Dominant symptom score (VAS)
- Progression-free survival (symptom control)
- Overall survival
- Acute toxicity (RTOG/CTCAE v.4.03)
- Quality of life (EORTC-QLQ-C30)
- Change in ADC from baseline to end of treatment
- Change in ADC from baseline to 6 months following treatment

### Study design

This is an international, multi-center single arm prospective feasibility study.

### Inclusion criteria

- Patients with hormone refractory biopsy proven HRPC
- Presenting with a dominant debilitating symptom
- Expected median survival of at least 3 months [5]
- Focal irradiation of lesion is feasible
- Systemic therapy according to guidelines
- Age ≥ 18 years
- Legal capacity, patient understands the nature, significance and consequences of the study.
- Written informed consent

### Exclusion criteria

- Relevant comorbidity (with limitations to administer radiotherapy according to protocol)
- Prior radiotherapy which results in limitations to administer radiotherapy according to protocol
- No large metal implants in vicinity of lesion
- Department dose constraints for normal tissue can’t be met
- Large bony lesions with extensive osseous destruction (e.g. vertebral body)
- Patients symptoms do not correlate with MRI findings

### Treatment

#### Systemic therapy

Systemic therapy will be administered according to department standard guidelines. Systemic therapy may be chemotherapy, anti-hormonal therapy, targeted therapies, bisphosphonates or a combination. The type of therapy will be documented. Interruptions of systemic therapy, delays or changes of drugs due to radiotherapy should be avoided.

#### Radiotherapy

Preferred technique is hypo-fractionation with 4 fractions of high-precision radiotherapy (SBRT, SRS or VMAT). The dominant symptomatic lesion will receive 4 × 5 Gy of high-precision radiotherapy, and the most aggressive part of the lesion 4 × 7 Gy using a simultaneous integrated boost (SIB) technique. Daily IGRT is mandatory for hypo-fractionated regimens. For the treatment of thoracic lesions and metastases in the upper abdomen, techniques for motion compensation (e.g. breath-hold or gating techniques) should be used, if available. For specific situations (e.g. spinal metastases with involvement of the spinal canal or cord compression or metastases with infiltration of hollow organs), lower total doses and lower doses per fraction may be required for adherence to dose constraints and avoidance of radiation-induced complications such as perforation. In such situations, moderate hypo-fractionation with more than 4 fractions and doses per fraction < 5 Gy is allowed. The total dose should be equivalent to at least 50 Gy of conventional fractionation.

A radiologist will review the MRI scans to validate correspondence between the lesion on MRI and the patient’s dominant symptom. In case of more than one lesion, decision on priority of target volumes will be made by the radiation oncologist based on clinical expertise. An ADC map will be calculated for the lesion using the DWI sequences.

The dominant symptomatic lesion (GTV1) is drawn manually by a radiologist and a radiation oncologist using the information from T2w-MRI and computed tomography (CT) scans [27]. The most aggressive part of the dominant lesion (GTV2) is defined by using the ADC map. An auxiliary volume is created including only voxels in the GTV1 that presents with ADC values below 1200 × 10^− 6^ mm^2^/s. This is necessary, because the GTV1 drawn by the clinicians may contain benign tissue with relatively large ADC values. The median ADC value for the auxiliary volume is then calculated. The most aggressive part of the tumor is then defined as voxels with an ADC value below this median ADC value. Notice that GTV2 need not to be one coherent volume, but may consist of several sub-volumes. In this study, the clinical target volume (CTV) is the same as the GTV (visible macroscopic tumor). The safety margin for the planning target volume (PTV) should be according to the department standard, and depending on the radiation technique used. The margin for the PTV2 for CTV2 may be chosen to be smaller than for PTV1as this volume is enclosed in the CTV1, which receives a dose of 4 × 5 Gy already. For the treatment of lesions moving according to breathing (e.g. in the upper abdomen), treatment techniques with motion compensation (e.g. breath-hold or gating) should be used.

### Assessments

Patients are scheduled for follow-up visits at 1, 3, 6 months after end of radiotherapy. Additional MRI (T2w and DWI) will be performed at 1 and 6 months after end of radiotherapy or in case of progression of the dominant symptom. A complete staging according to current guidelines with adequate imaging is required. The available data should give sufficient information about:

- Number of visible metastatic lesions
- location of each lesion (e.g. lung metastasis in left lower lobe, osseous metastases in the sacrum)
- Size of each lesion

### Baseline examination

A baseline diagnostic MRI corresponding to dominant symptom is required, and the MRI-scans must be co-registered to imaging necessary for radiotherapy planning CT. CT is allowed for judgement of the stability of bone metastases in addition to MRI. However, MRI (diffusion weighted) is required to identify the most aggressive part of DSL, and T2w MRI is needed to co-register the diffusion weighted MRI to the planning CT.

Baseline examination includes:

- Review of medical history inclusive previous anti-cancer treatment
- Review of clinical routine blood and radiology results
- Review of concomitant medication, analgesia, opioids, current systemic anti-cancer treatment
- Tumor staging, grading, classification, localizations, sizes
- Physical examination (incl. Height, weight)
- Performance status (ECOG)
- Baseline dominant symptom (VAS)
- Baseline acute toxicity RTOG/CTCAE v.4.03)
- Quality of life (EORTC-QLQ C30)

### End of radiotherapy

- Review of concomitant medication, analgesia, opioids, current systemic anti-cancer treatment
- Review of clinical routine blood results and radiology
- Dominant symptom score (VAS)
- Acute toxicity (RTOG/CTCAE v4.03)
- Performance status (ECOG)
- Assessment of AEs/SAEs
- Radiotherapy techniques used
- Volumes, fractions, doses applied, dose constraint compliance for all organs at risk
- Cumulative doses to target volumes, any boosts

### Follow-up visits after radiotherapy

Follow-up visits will be performed at 1, 3 and 6 months after the end of radiation treatment including the following items:

- Review of concomitant medication, analgesia, opioids, current systemic anti-cancer treatment
- Review of clinical routine blood and radiology results
- Dominant symptom score (VAS)
- Acute toxicity (RTOG/CTCAE v4.03)
- Quality of life (EORTC-QLQ C30) (at 6 months only)
- Performance status (ECOG)
- Assessment of AEs/SAEs

### Diagnostic procedures during the follow-up period

Protocol specific follow-up MRI scans are required for patient visit at one and six months after end of radiotherapy or at progression of the dominant symptom. Remission status evaluated at 6 month based on available clinical data. Follow-up MRI scans will be co-registered to the baseline MRI scans. Comparative volume and ADC statistics will be calculated as surrogate markers of response to radiotherapy.

### Sample size calculation

The objective of this study is to investigate the feasibility of applying hypo-fractionated radiotherapy to treat a dominant symptomatic lesion in patients with HRPC. The hypothesis is that at least 90% of the recruited patients complete combined systemic and local treatment with at least 90% of scheduled radiation dose administered. We use a two-sided one-sample proportion score test to compare the proportion of feasible cases to a reference value of 90%. We want to detect any difference with statistical significance of 5% and a power of 80% for not overlooking a true proportion non-feasible of 70%. The necessary sample size is estimated to 24 cases. Drop-out may happen either due to exclusion, or because patients withdraw their consent for participation. For patients with poor prognosis a realistic drop-out rate is estimated to be 30%, which implicated that 34 patients must be included in the study to reach significance.

## Discussion

Patients with metastatic PC have a better survival prognosis when compared to patients with metastases from several other solid tumors and may live for several months or even years [28, 29]. Thus, a considerable number of these patients may live long enough to experience treatment-related late toxicities and a recurrence of the treated lesions. Many of these patients receive radiotherapy, which needs to consider several aspects including symptom control, ideally for the patients’ remaining lifetime, and its feasibility [6–9]. Since PC requires higher radiation doses than many other solid tumors, the goal of delivering a high and safe dose often may not be possible with conventional RT. This problem may be solved with modern high-precision RT techniques such as SBRT, SRS and VMAT that generally allow a better sparing of the surrounding organs at risk and/or an escalation of the dose to the metastatic lesions [20, 30]. Moreover, since metastatic PC is an incurable situation, the overall treatment time of radiotherapy should be kept as short as possible. Therefore, hypo-fractionated high-precision RT with very few fractions appears a reasonable option. In a palliative situation like metastatic PC, control of debilitating symptoms is very important, and often the most important goal for the patients [6, 7, 9]. Thus, one may consider focusing the treatment on the symptomatic lesions in order to avoid unnecessary toxicity by treating very large volumes and/or many sites. Since high-precision radiotherapy techniques are both resources and time consuming, the patients to be included in this study must have an expected survival prognosis of at least 3 months.

Therefore, the present study protocol has been developed that combines different aspects of radiotherapy for metastatic PC, namely high-precision RT with very few fractions focusing on the dominant symptomatic lesion. In order to deliver a high biologically effective dose allowing for long-term symptom control without increasing the risk of treatment-related toxicity, an additional SIB is administered to the most aggressive part of the dominant lesion. The present study investigates the feasibility of the novel approach, as well as response, symptom control, overall survival, acute toxicity and quality of life.

If this new radiotherapy regimen proves to be feasible, a prospective randomized phase II/III dose escalation study will be designed to contribute to the improvement of the outcomes of palliative radiotherapy of symptomatic metastatic HRPC in terms of better long-term symptom control and less radiation-related toxicity.

## Abbreviations

ADC: apparent diffusion coefficient
AE: adverse event
CT: computed tomography
CTCAE: Common Terminology Criteria for Adverse Events
CTV: clinical target volume
DSL: dominant symptomatic lesion
DWI: diffusion weighted imaging
ECOG: Eastern Cooperative Oncology Group
EORTC: European Organisation for Research and Treatment of Cancer
GTV: gross tumor volume
HRPC: hormone-refractory prostate cancer
IGRT: image-guided radiotherapy
MRI: magnetic resonance imaging
PC: prostate cancer
PTV: planning target volume
QLQ: quality of life questionnaire
RT: radiotherapy
RTOG: Radiation Therapy Oncology Group
SAE: serious adverse event
SBRT: stereotactic body radiation therapy
SIB: simultaneous integrated boost
SRS: stereotactic radiosurgery
VAS: visual analogue scale
VMAT: volumetric modulated arc therapy
